# LH level on the antagonist administration day as a predictor of the reproductive outcomes in women with normal ovarian function

**DOI:** 10.3389/fendo.2023.1232361

**Published:** 2023-09-19

**Authors:** Conghui Pang, Kehua Wang, Ruihan Wang, Danyang Guo, Zixi Wen

**Affiliations:** ^1^ The First Clinical College, Shandong University of Traditional Chinese Medicine, Jinan, Shandong, China; ^2^ Department of Reproduction and Genetics, The Affiliated Hospital of Shandong University of Traditional Chinese Medicine, Jinan, Shandong, China

**Keywords:** GnRH antagonist protocol, LH level, antagonist administration day, laboratory indicators, pregnancy outcomes

## Abstract

**Introduction:**

The addition of antagonists is mainly based on estrogen level and follicle size, while LH level has not received sufficient attention.In this study, LH Level on the antagonist administration day was used as the main research objective to explore its relationship with laboratory indicators and pregnancy outcomes.

**Methods and Analysis:**

We enrolled 854 patients with normal ovarian function undergoing *in-vitro* fertilization (IVF) or intracytoplasmic sperm injection (ICSI) between May 2021 to May 2022 at the Reproductive Center of Shandong University of Traditional Chinese Medicine.We used the quartile method to group LH levels on the antagonist administration day. There were four groups: Q1 (0.53IU/L≤LH ≤ 1.89IU/L); Q2 (1.89IU/L<LH ≤ 3.01IU/L); Q3 (3.01IU/L<LH≤ 5.29 IU/L); Q4 (5.29IU/L<LH ≤ 8.72IU/L). A total of 452 fresh embryo transplantation cycles and 1726 Frozen embryo transplantation cycles were carried out.

**Result:**

There were significant differences among the four groups in terms of total Gn dosage, E2, P and LH on trigger day, number of retrieved oocytes, number of 2PN embryos, number of blastocysts, Number of ET and fresh ETR.There is a significant correlation between LH on antagonist administration day and Basal LH Level,LH on trigger day,number of oocytes retrieved,number of 2PN embryos,number of blastocysts, number of ET.Using Fresh ETR,Fresh CPR,OHSS and Cumulative CPR as the criterion respectively, the optimal cut-off value for evaluating LH on antagonist administration day was 4.18IU/L,3.99IU/L,4.63IU/L,4.66IU/L.

**Conclusion:**

There was a significant positive correlation between LH on the antagonist administration day and number of oocytes retrieved,number of 2PN embryos,number of blastocysts.LH on the antagonist administration day could predict Fresh CPR,OHSS and Cumulative CPR to some extent.

## 1 Introduction

In recent decades, *in vitro* fertilization-embryo transfer (IVF-ET) has grown rapidly throughout the world, becoming an important method of treating infertility. The treatment process revolves around controlled ovarian hyperstimulation (COH). Gonadotropin-releasing hormone antagonist (GnRH-ant) protocols are widely used due to their advantages of short stimulation time, low costs, and a lower incidence of ovarian hyperstimulation syndrome (OHSS) (1–3). GnRH-ant binds to specific receptors on the pituitary gland and inhibits endogenous Luteinizing hormone (LH). It can prevent the appearance of early follicular LH surge, thereby inhibiting premature follicle production and reducing the cycle cancellation rate, which brings a new choice for clinical ovulation induction programs (4).

LH is a glycoprotein hormone secreted by the pituitary gland, which plays an important role in estrogen synthesis, follicle development, and ovulation induction (5).On the one hand, high LH levels are harmful to pregnancy outcomes in both the natural and ovarian stimulation cycles. Too little LH, on the other hand, is linked to pregnancy loss (6, 7). Scholars generally agree that an adequate level of LH is required for follicular development. According to some studies, the LH window has a range of 1.2–5 IU/L (8, 9). The team led by Professor Li Yuan proposed that LH≥4IU/L be considered the critical value, and antagonists should be considered when the threshold was exceeded (10). However, there is no consensus on the appropriate value of LH during COH with antagonist protocol.

At present, antagonist protocols are mainly divided into fixed and flexible protocols (11). The starting day of GnRH antagonist administration (i.e., both the fixed and flexible protocols) is mainly based on the day of ovarian stimulation, the diameter of the follicles, the estradiol levels, or a combination of these parameters (3). However, LH levels on the antagonist administration day have received less attention.

A retrospective analysis was used in this study. We divided the LH level on the antagonist administration day into four groups according to the quartile method and compared the laboratory indicators and pregnancy outcomes among the four groups.

Statistical methods were used to analyze the effects of LH level on the antagonist administration day on laboratory indicators and estimate the cut-off values of LH on antagonist administration day for predicting various pregnancy outcomes,so as to illustrate that the LH Level on the antagonist administration day could be used as a predictor of the reproductive outcomes in women with normal ovarian function.

## 2 Materials and methods

### 2.1 Participants

**Figure 1:**
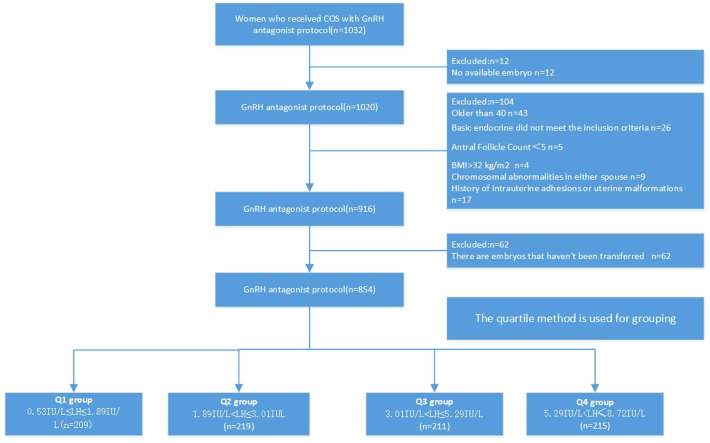
A flow chart describing the GnRH-ant protocol in all patients.

Through a database search, the data of patients who underwent IVF/ICSI cycles in the Affiliated Hospital of Shandong University of Traditional Chinese Medicine from May 2021 to May 2022 were selected. Only those infertile patients who received the GnRH-ant regimen to generate usable embryos and had all embryos transferred were included. All enrolled patients signed informed consent.This study was approved by the Reproductive Medicine Ethics Committee of the Affiliated Hospital of Shandong University of Chinese Medicine(No.20210713).The patients’ flow chart detailing the whole process is shown in **Figure 1** 3mL of fasting elbow venous blood was collected and plasma LH, FSH and E2 levels were detected by luteinizing hormone assay kit, follicle-stimulating hormone assay kit and estradiol assay kit (Beckman Coulter, Inc, USA).

Inclusion criteria were: patients aged 20–40 years; patients with body mass index (BMI) ≤32 kg/m^2^; basal follicle-stimulating hormone (FSH) ≤10mIU/mL; normal thyroid stimulating hormone (TSH) and prolactin levels as well as patients with no preconditioning with oral contraceptives.

Exclusion criteria were: recurrent abortion or chromosomal abnormalities; patients with a history of uterine malformations and intrauterine adhesions;woman with PCOS diagnosed according to Rotterdam criteria (12);Poor responders identified according to Bologna criteria (13);Presence of clinically significant systemic diseases or other endocrine diseases.

### 2.2 Protocol for controlled ovarian stimulation

Ovarian stimulation began on days 2 or 3 of the menstrual cycle with recombinant FSH (150–450 IU) (Gonal-F, Merck Serono, Coinsins, Switzerland) daily with or without 75–300 IU of human menopausal gonadotropin (hMG, Livzon, Shanghai, China). Moreover, hMG was used in patients where a poor response was anticipated because of advanced age, low antral follicle count.The starting dose of Gn (FSH/hMG) was based on the patient’s age, BMI, antral follicle count(AFC), and hormonal profile. The doses were adjusted according to serum estradiol (E2) level and ovarian response, which was evaluated by transvaginal ultrasound. The administration of GnRH-ant, Ganirelix, or Cetrotide (0.25 mg daily at10:00 AM) was started either on the 6th day of recombinant FSH stimulation until the hCG injection or when the dominant follicle’s diameter was ≥ 12–14 mm or estrogen level >250pg/mL.

After the three follicles reached a mean diameter of 17 mm, or two follicles were over 18 mm, final oocyte maturation was triggered by administering recombinant human chorionic gonadotropin (rhCG, 250 µg, Merck Schlano, Germany) or Decapeptyl (0.2 mg) either alone or in combination with urinary hCG (2000 IU, Livzon, China)When a patient was suspected to be at risk for ovarian hyperstimulation syndrome. After 35 to 37 h, the eggs were harvested by transvaginal ultrasound.

### 2.3 Embryo transfer and luteal support

On the 3rd to 5th day after fertilization, 1–2 embryos of high-quality were selectively transferred. Embryo grading was done in accordance with the proceedings of the Istanbul consensus (14). High-quality embryos in our center were defined as having 6-10 blastomes on the third day, basically uniform size of blastomes, and fragmentation rate ≤20%.We divided blastocysts into 1-6 stages according to the degree of blastocyst expansion and incubation,the quality of inner cell mass (ICM) and trophoblast cell (TE) was further evaluated for the blastocyst of stage 3-6.Blastocysts with scores ≥ 3BB were defined as high quality blastocysts.

The luteal phase support was started on the day of oocyte retrieval with intramuscular progesterone injections (20 mg, Xian Ju Pharmaceutical Co, China) twice a day. Additionally, dydrogesterone (20 mg, Abbott Laboratories, USA) was taken twice each day.

### 2.4 FET protocol

At least one of the above three features must be present,the patient underwent frozen-thawed embryo transfer (FET).①E2 on trigger day≥5000pg/ml;②E2 on trigger day was between 4000 and 5000 pg/ml,number of oocytes retrieved was between 15 and 20, but the patient has symptoms such as bloating;③number of oocytes retrieved ≥20.

### 2.5 Outcome measures

The primary outcome measure was the Clinical pregnancy rate of fresh embryo transfer(fresh CPR) and all embryo transfer cycles(Cumulative CPR). These secondary outcomes included the number of retrieved oocytes, the number of high-quality embryos, the fresh embryo transfer rate (fresh ETR), the rate of Ovarian hyperstimulation syndrome (OHSS rate). Clinical pregnancy was defined as the confirmation of gestational sac and fetal heartbeats by transvaginal ultrasound 28 days after ET. Fresh CPR was the ratio of the number of pregnancy cycles after fresh-ET to the total number of fresh embryo transfer cycles. Cumulative CPR was the ratio of the number of clinical pregnancies following the transfer of all embryos from one ovulation cycle to the total number of ovulation cycles. Fresh ETR was defined as the ratio of fresh embryo transfer cycles to oocyte retrieval cycles. OHSS is defined by Golan et al. Standards (15).

### 2.6 Statistical analysis

Statistical software SPSS(version 26.0) was used for statistical analysis. Kolmogorov-Smirnov test was used to test whether continuous numerical variables obeyed normal distribution, If the data was distributed normally, it was expressed by mean and standard deviation.If continuous numerical variables do not follow the normal distribution, the data was represented by the median and upper and lower quartiles [M(P25, P75)], and the rank sum test was used for comparison.Counting data was described by n(%) and Chi-square test was used to compare the distribution differences between groups.If sample size > 40 and theoretical frequency > 5, Pearson Chi-square test was used for non-parametric test;If the sample size is less than 40 or the theoretical frequency is less than 5, Fisher’s exact probability method is used to test.a=0.05 was used as the test level, P<0.05 was considered statistically significant, And the cut-off value of Yoden index was calculated by ROC curve.

## 3 Results

A total of 1032 patients were included in the initial analysis, and 854 patients were included in the final study after applying the exclusion criteria. No cycle cancellation due to unexpected premature ovulation was reported among patients of groups.

Age, BMI, infertility type, infertility years, causes of infertility,basal FSH level, basal E2 level, basal T level, started Gn dose, time of antagonist administration, and fertilization method were not significantly different among the four groups. Basal LH levels were significantly different among the four groups. (**Table 1**).

**Table 1:** Analysis of demographic and clinical characteristics among the four groups.

Variables	Q1 group (n = 209)	Q2 group (n = 219)	Q3 group (n = 211)	Q4 group (n = 215)	F/H/X^2^	*P* value
Age (years)	33.07 ± 4.38	32.36 ± 4.26	33.17 ± 4.18	32.31 ± 4.29	2.404	0.066
BMI	24.69 ± 3.54	24.07 ± 3.39	24 ± 3.48	23.93 ± 3.52	2.085	0.101
Infertility type, n (%)					5.713	0.126
Primary infertility	90 (43)	102 (47)	75 (36)	93 (43)		
Secondary infertility	119 (57)	117 (53)	136 (64)	122 (57)		
Infertility years (years)	3 (2, 4)	3 (2, 4)	3 (2, 4.5)	3 (2, 4)	2.607	0.456
Causes of infertility					1.326	0.97
Tubal factor	178 (85)	185 (84)	175 (82)	180 (84)		
Male factor	29 (14)	31 (14)	35 (16)	32 (15)		
Tubal factor and Male factor	2 (1)	3 (2)	4 (2)	3 (1)		
Basal E2 Level (pg/mL)	45.95 ± 11.15	46.15 ± 8.96	46.91 ± 10.04	46.94 ± 9.64	0.566	0.637
Basal FSH Level (IU/L)	7.13 ± 1.76	7.04 ± 1.34	7.07 ± 1.41	6.77 ± 1.29	2.449	0.062
Basal LH Level (IU/L)	4.29 ± 1.43	4.68 ± 1.55	5.18 ± 1.68	5.75 ± 1.9	31.119	< 0.001
Basal T Level (µg/L)	0.39 ± 0.12	0.38 ± 0.1	0.37 ± 0.11	0.39 ± 0.11	1.29	0.276
Started Gn dose(IU)	224.46 ± 41.64	218.88 ± 39.27	217.71 ± 40.21	214.01 ± 38.64	2.481	0.06
Time of antagonist administration (D)	5.77 ± 1.1	5.74 ± 1.09	5.85 ± 1.11	5.84 ± 0.93	0.572	0.633
Fertilization method (n,%)					2.141	0.544
IVF	170 (81)	172 (79)	176 (84)	177 (84)		
ICSI	39 (19)	45 (21)	34 (16)	34 (16)		

Laboratory indicators and pregnancy outcomes were compared among the four groups. There were no significant differences among the four groups in terms of duration of Gn, endometrium on trigger day, number of embryos (D3),number of high-quality embryos,fresh CPR,OHSS rate and Cumulative CPR.There were significant differences among the four groups in terms of total Gn dosage, E2 on trigger day, P on trigger day,LH on trigger day, number of retrieved oocytes, number of 2PN embryos, number of blastocysts, Number of ET and fresh ETR. (**Table 2**).

**Table 2:** Comparison of laboratory indicators and pregnancy outcomes among four group.

Variables	Q1 group (n = 209)	Q2 group (n = 219)	Q3 group (n = 211)	Q4 group (n = 215)	F/H/X^2^	*P* value
Duration of Gn(d)	9.53 ± 1.58	9.21 ± 1.56	9.34 ± 1.63	9.31 ± 1.68	1.431	0.232
Total dosage of Gn(IU)	2250 (1800, 2700)	2000 (1620, 2475)	2025 (1800, 2400)	1925 (1575, 2400)	22.692	< 0.001
E2 on trigger day (pg/mL)	2056 (1386, 2886)	2194 (1450.5, 3685)	2729 (1727, 3972.5)	3330 (1980, 4924.5)	52.142	< 0.001
P on trigger day (nmol/L)	1.08 (0.73, 1.49)	1.08 (0.78, 1.54)	1.13 (0.8, 1.48)	1.25 (0.84, 1.77)	9.762	0.021
LH on trigger day (IU/L)	1.82 (1.05, 2.83)	1.98 (1.35, 2.81)	2.67 (1.83, 4.54)	3.14 (1.79, 5.44)	74.62	< 0.001
Endometrium on trigger day(cm)	1.18 (1, 1.33)	1.16 (0.97, 1.31)	1.15 (1, 1.29)	1.15 (0.98, 1.28)	3.017	0.389
Number of oocytes retrieved	9 (7, 12)	10 (6.5, 13)	10 (6, 14)	12 (7, 17)	11.888	0.008
Number of 2PN embryos	6.53 ± 2.96	6.92 ± 3.47	7.16 ± 3.69	7.56 ± 4.2	3.012	0.029
Number of embryos(D3)	4 (2, 4)	4 (2, 4)	3 (2, 4)	3 (2, 4)	2.097	0.552
Number of blastocysts	0 (0, 1)	1 (0, 2)	1 (1, 2.5)	1 (1, 3)	147.658	< 0.001
Number of high-quality embryos	1 (0, 2)	1 (0, 2)	1 (0, 2)	1 (0, 2)	0.567	0.904
Number of ET	2 (0, 2)	2 (0, 2)	2 (0, 2)	0 (0, 2)	14.241	0.003
Fresh ETR, n (%)	124 (59)	122 (56)	114 (54)	92 (43)	13.088	0.004
Fresh CPR, n (%)	46(37)	52(43)	46(40)	48(52)	2.042	0.564
OHSS rate, n (%)	1(0)	1(0)	1(0)	2(1)	Fisher	0.94
Cumulative CPR, n (%)	160(77)	170(78)	170(81)	178(83)	3.142	0.37

**Figure 2:**
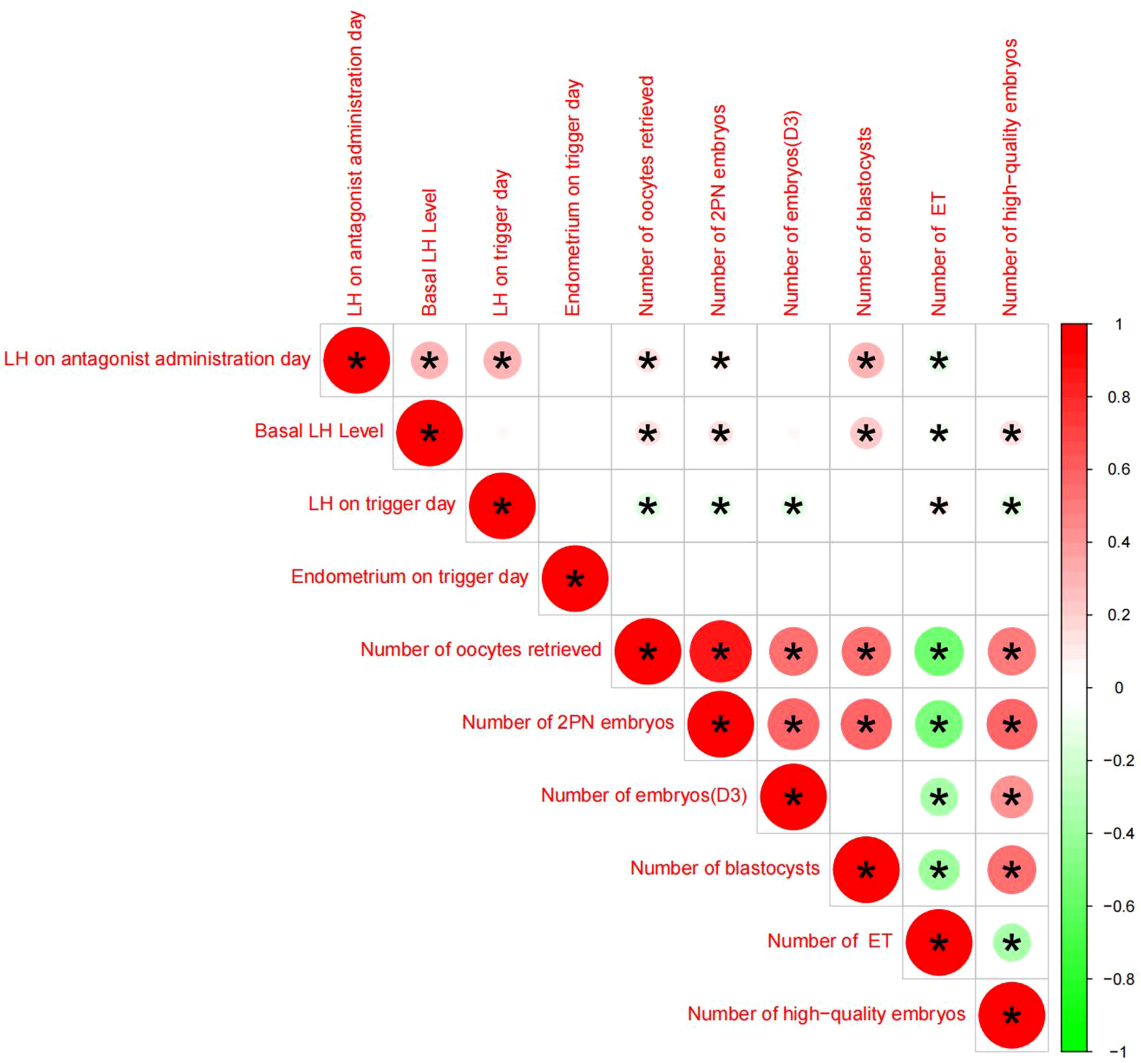
Correlation analysis between LH on the antagonist administration day and other indicators, An asterisk between the two indicators indicates statistical significance (there is a significant correlation),Red means positive correlation, green means negative correlation.

**Figure 3:**
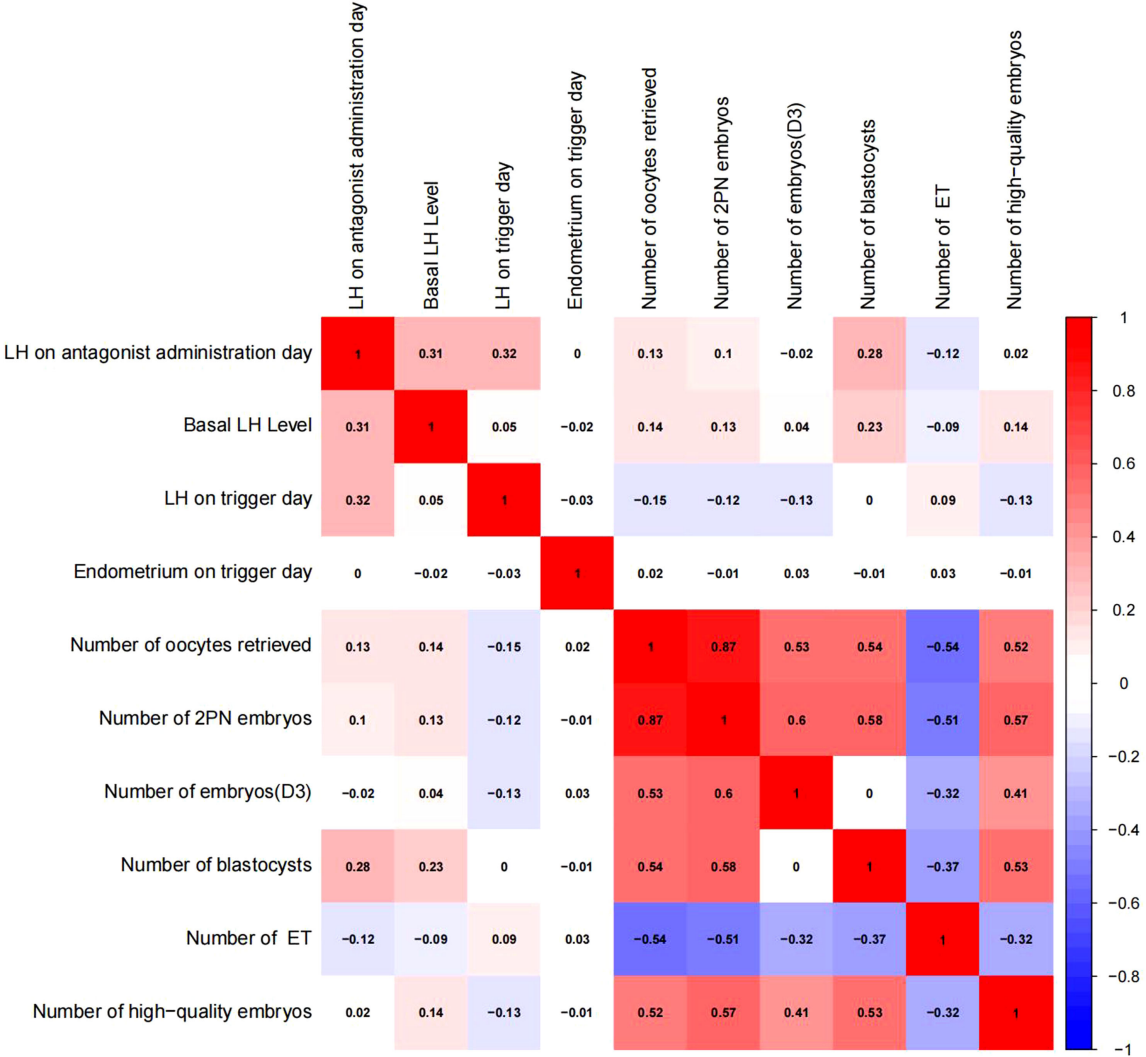
Correlation analysis between LH on the antagonist administration day and other indicators.The value between indexes represents the correlation coefficient R,R>0 means positive correlation, R < 0 means negative correlation.

Pearson chi-square test was used to verify the correlation between LH on antagonist administration day and basal LH level,LH on trigger day,endometrium on trigger day,number of oocytes retrieved,number of 2PN embryos,number of embryos (D3),number of blastocysts,number of ET and number of high-quality embryos.The results showed that LH on antagonist administration day had a significant correlation with basal LH level,LH on trigger day,number of oocytes retrieved,number of 2PN embryos,number of blastocysts,number of ET. (**Figures 2**, **3**)

**Figure 4:**
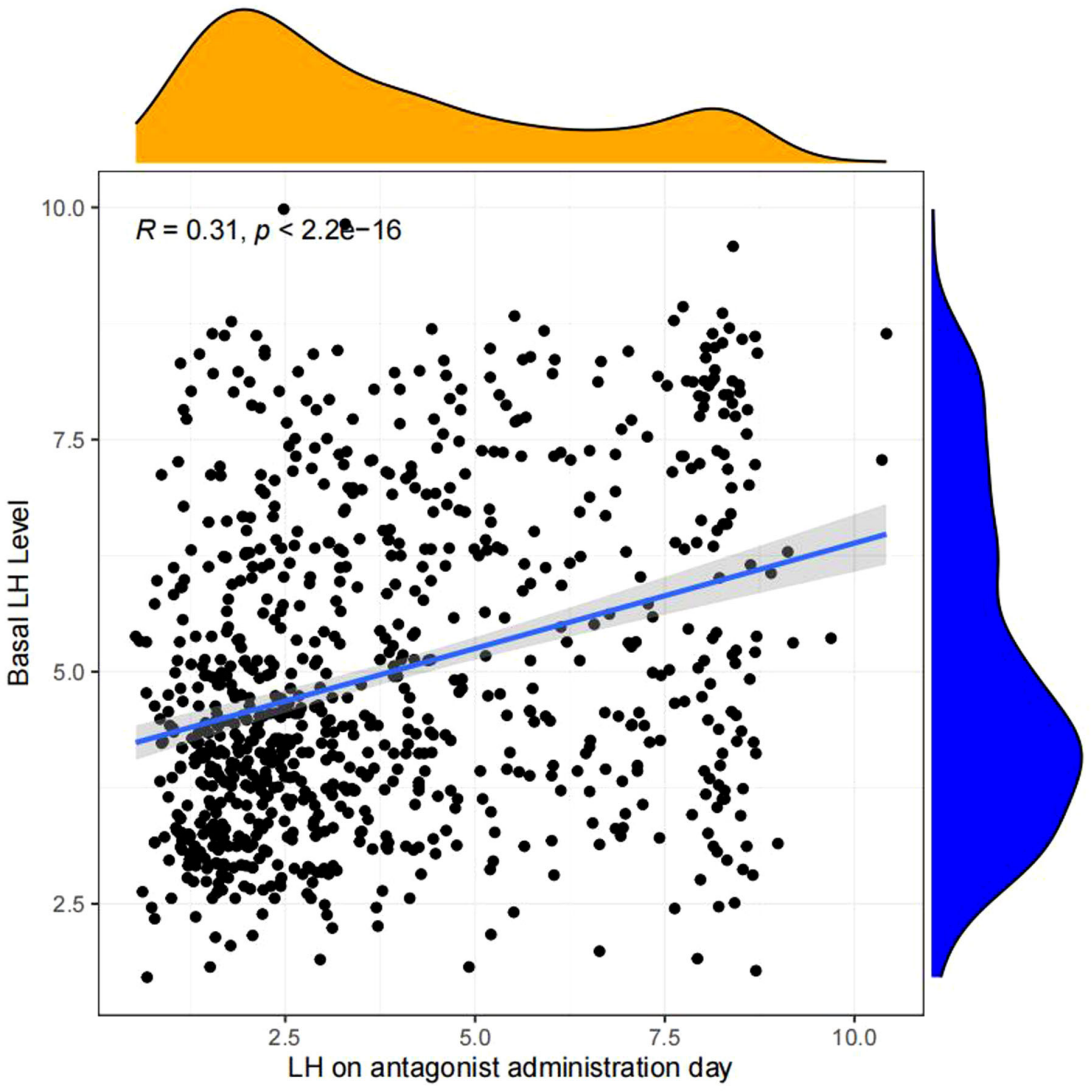
Linear correlation between LH on antagonist administration day and basal LH level.

**Figure 5:**
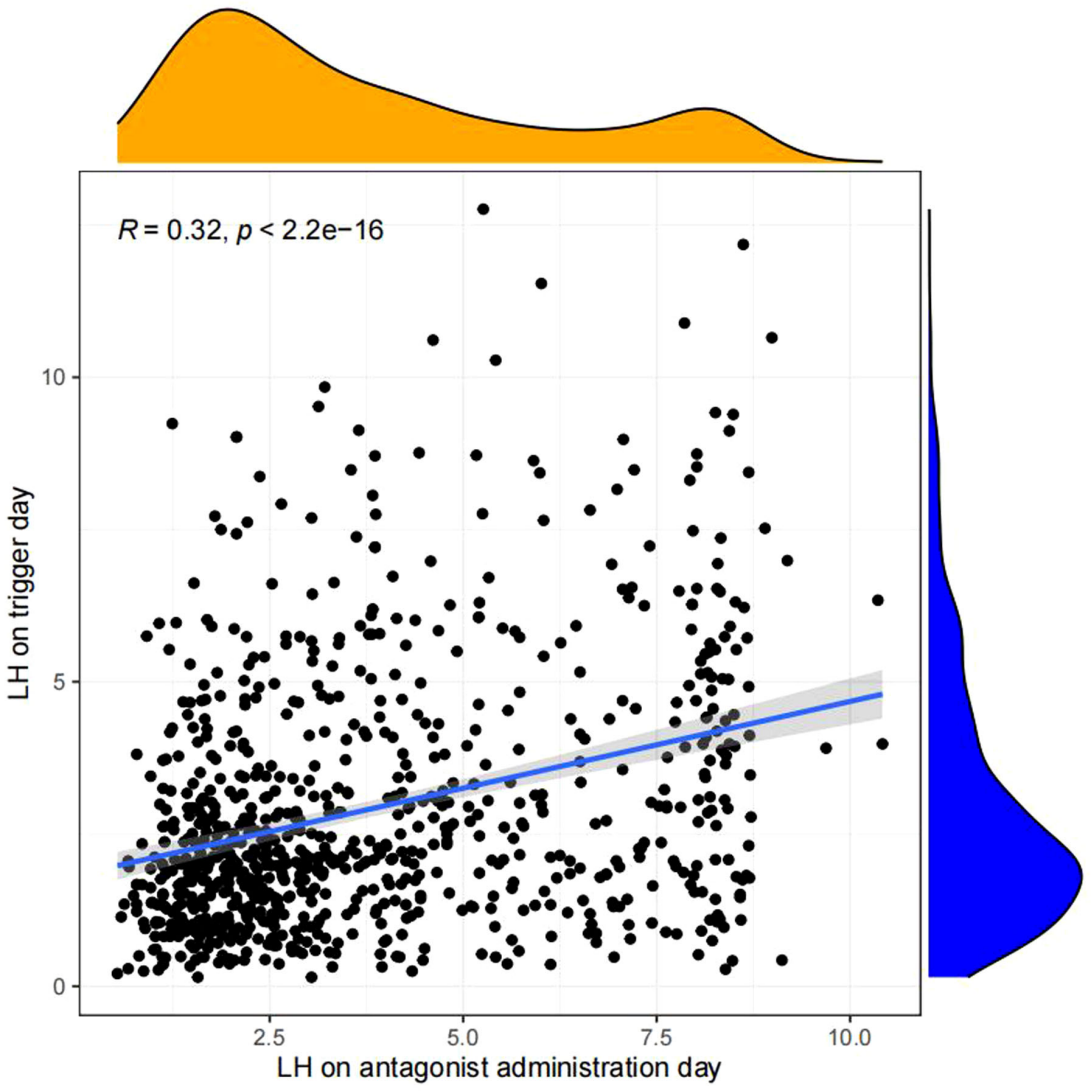
Linear correlation between LH on antagonist administration day and LH on trigger day.

**Figure 6:**
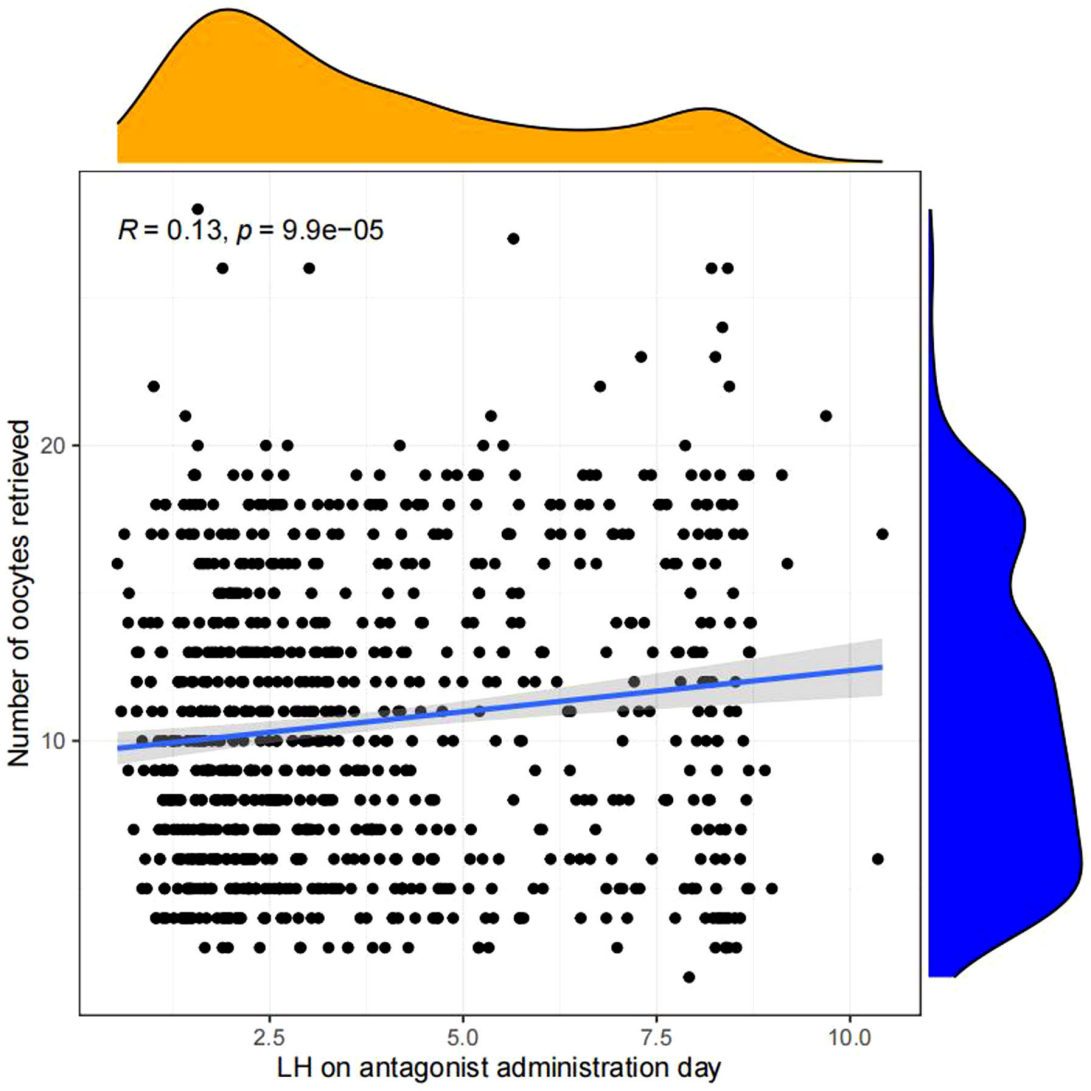
Linear correlation between LH on antagonist administration day and number of oocytes retrieved.

**Figure 7:**
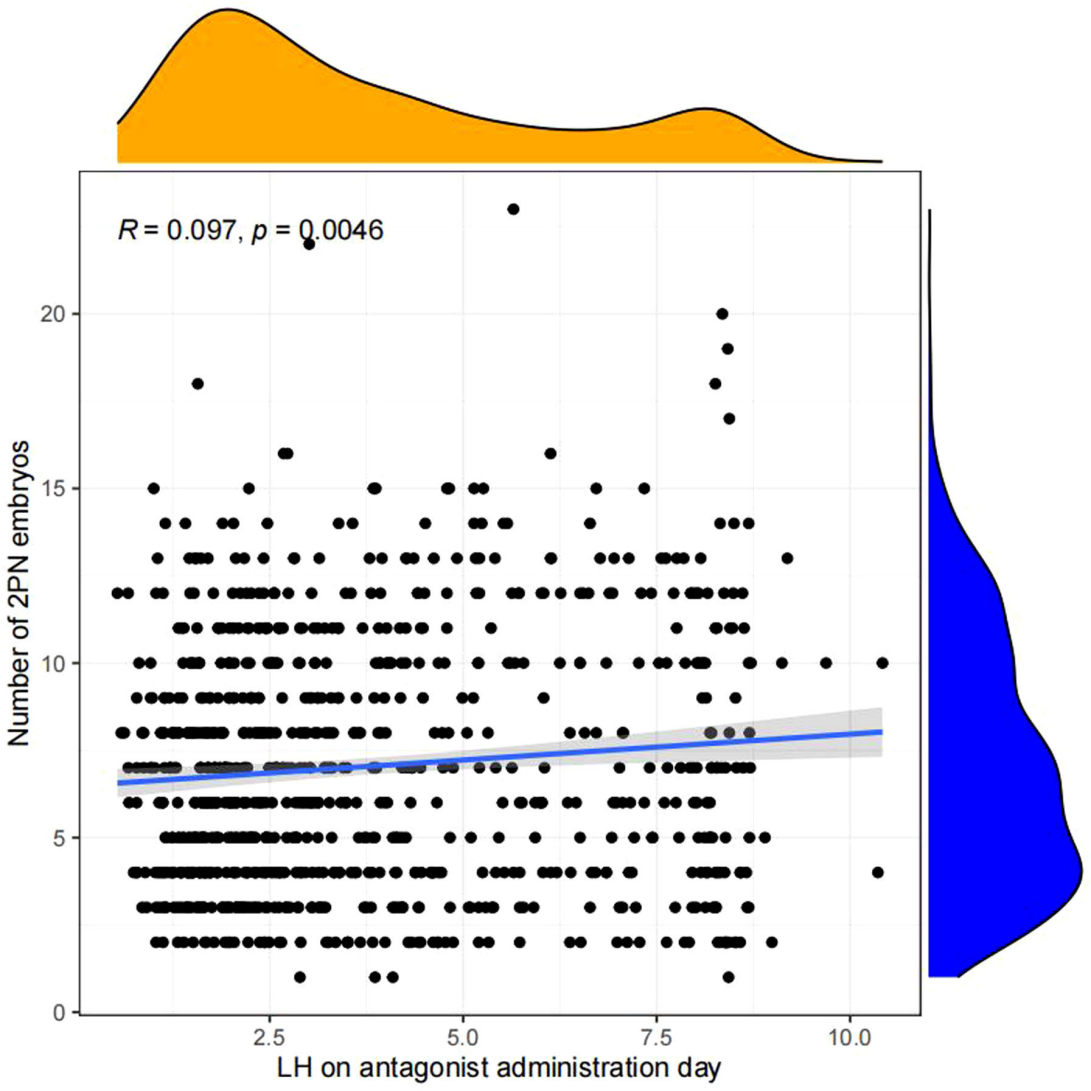
Linear correlation between LH on antagonist administration day and number of 2PN embryos.

**Figure 8:**
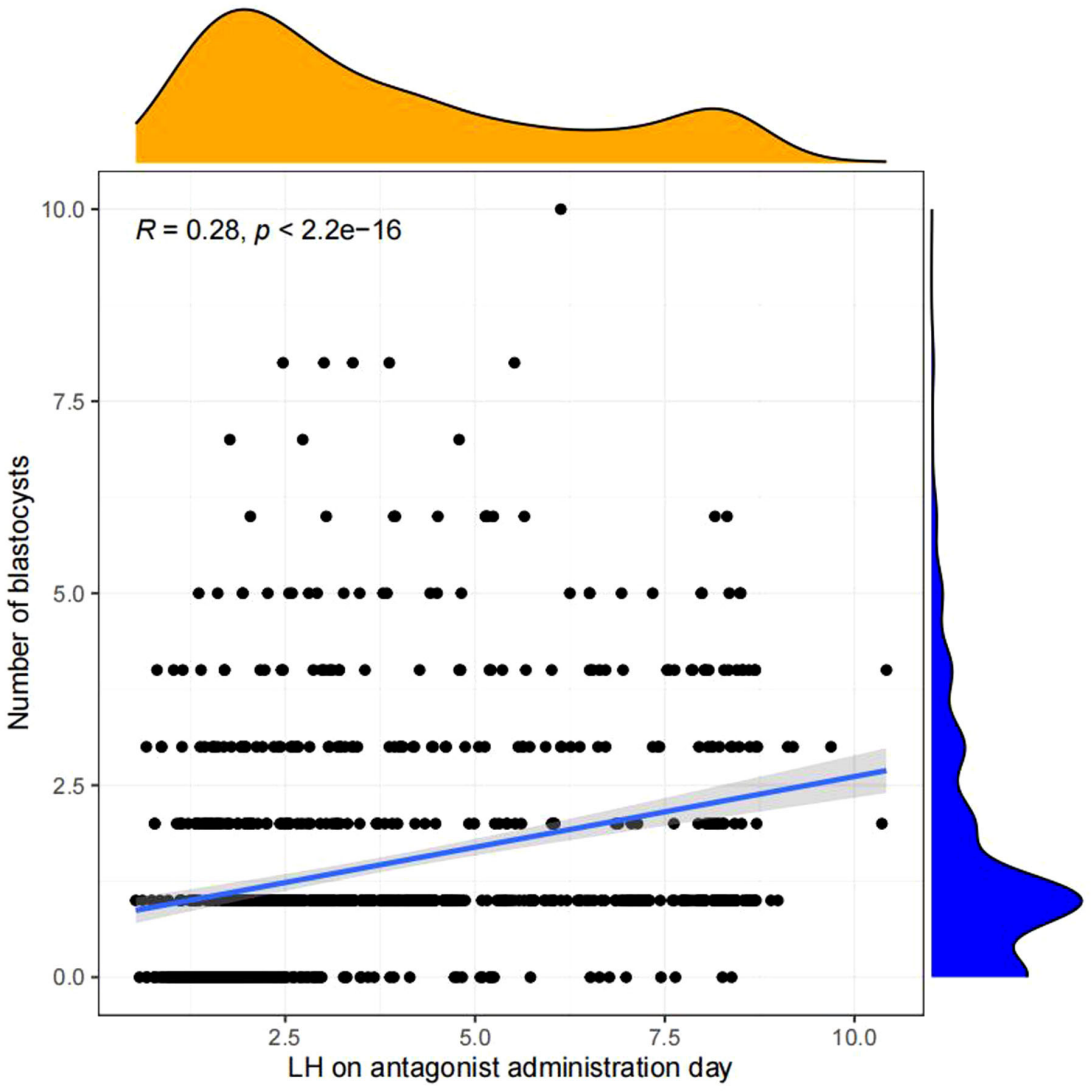
Linear correlation between LH on antagonist administration day and number of blastocysts.

**Figure 9:**
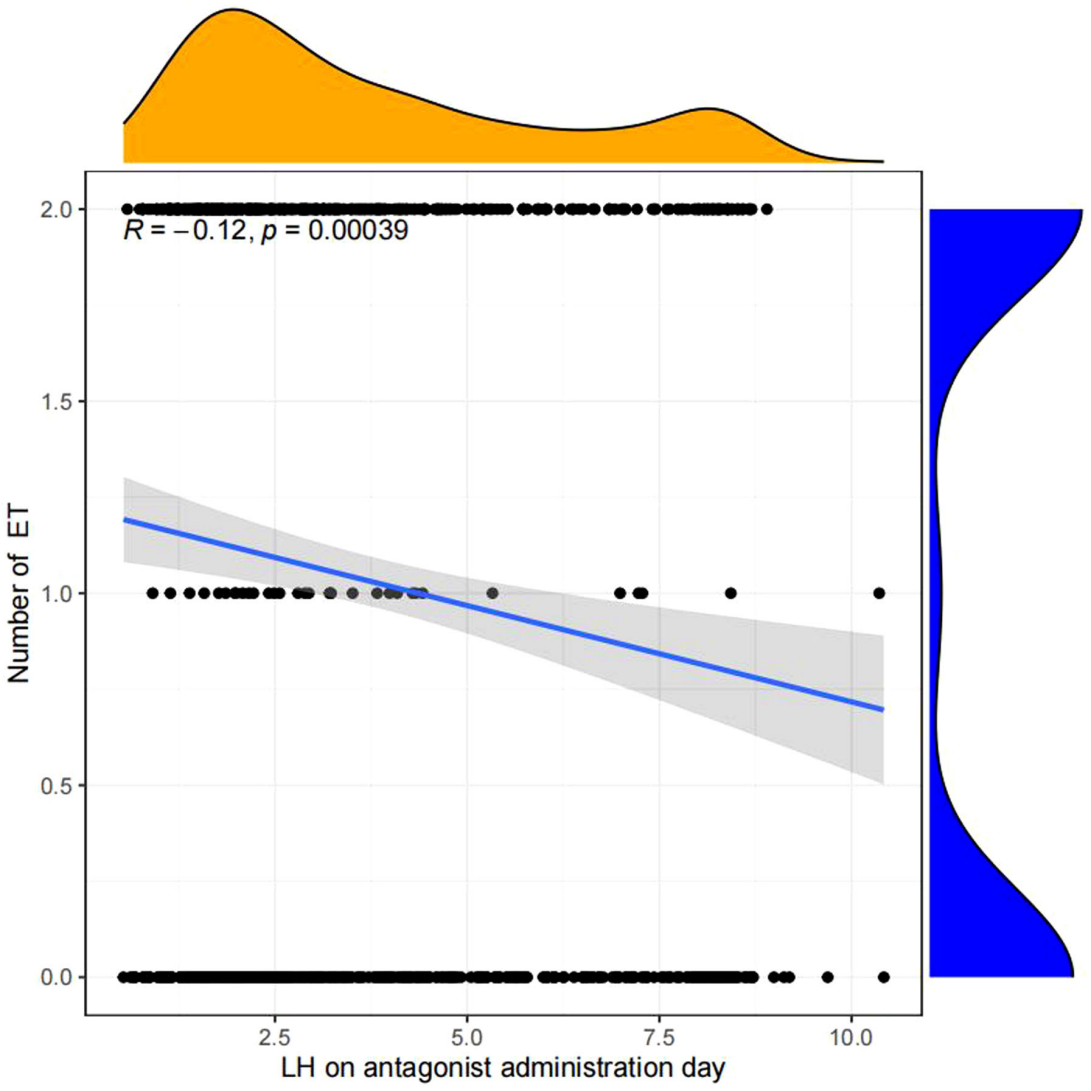
Linear correlation between LH on antagonist administration day and number of ET.

Linear regression analysis revealed that there is a significant positive correlation between LH on antagonist administration day and basal LH level (p<0.05, **Figure 4**), LH on trigger day (p<0.05,**Figure 5**), number of oocytes retrieved (p<0.05, **Figure 6**), number of 2PN embryos (p<0.05, **Figure 7**), number of blastocysts (p<0.05,**Figure 8**), number of ET (p<0.05, **Figure 9**).

**Figure 10:**
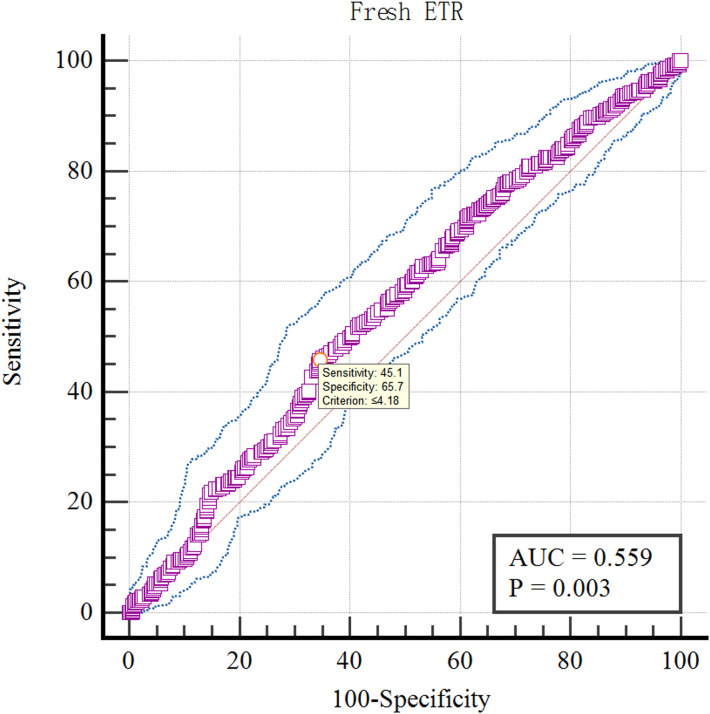
ROC curves of LH on antagonist administration day to predict Fresh ETR.

**Figure 11:**
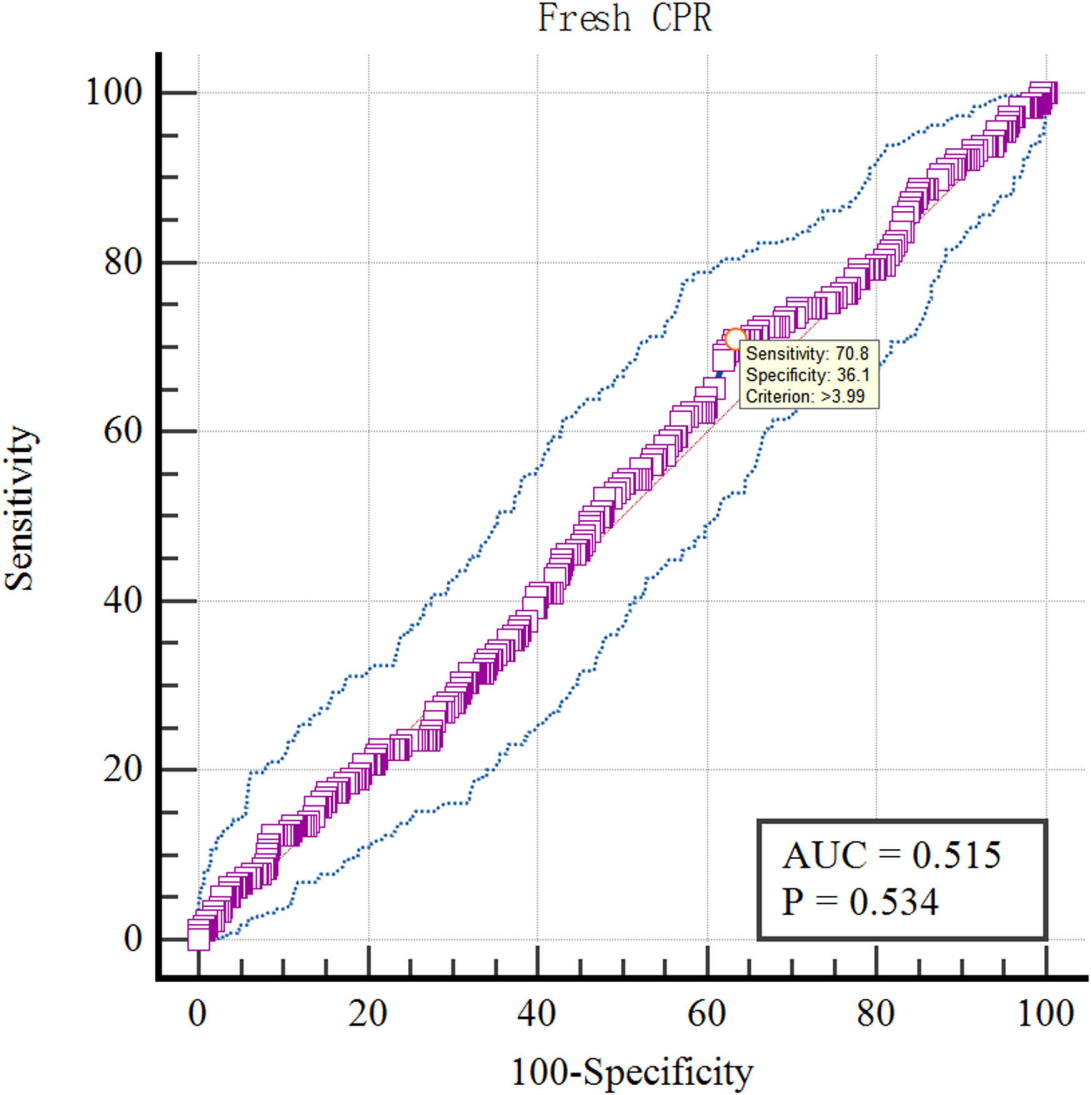
ROC curves of LH on antagonist administration day to predict Fresh CPR.

**Figure 12:**
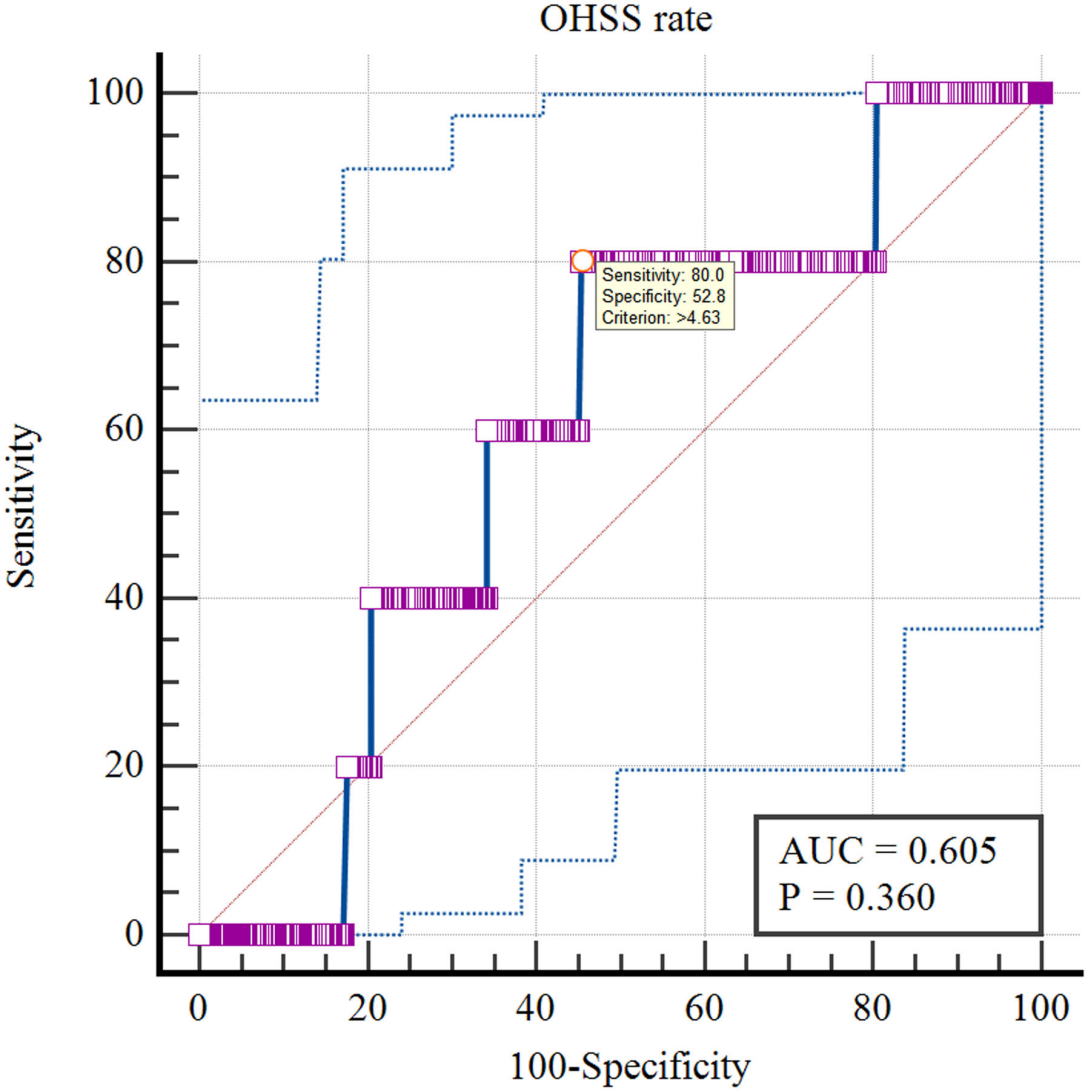
ROC curves of LH on antagonist administration day to predict OHSS.

**Figure 13:**
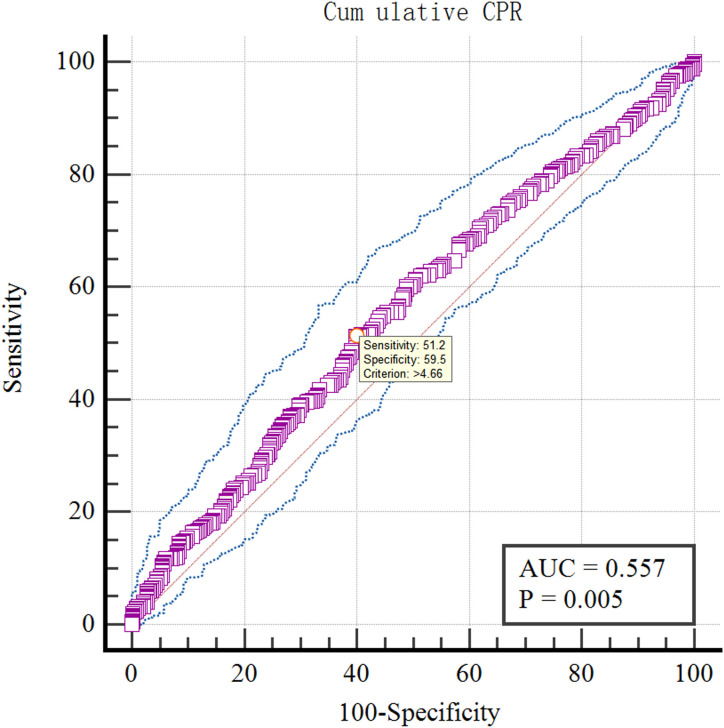
ROC curves of LH on antagonist administration day to predict Cumulative CPR.

The optimal cut-off value of LH on antagonist administration day of various pregnancy rates was analyzed by ROC curve.The results showed that the optimal cut-off value of LH on antagonist administration day was 4.18IU/L using Fresh ETR as the standard (AUC=0.559; P=0.003; **Figure 10**). Using Fresh CPR as the criterion, the optimal cut-off value for evaluating LH on antagonist administration day was 3.99IU/L (AUC=0.515; P=0.534; **Figure 11**). Using OHSS as the criterion, the optimal cut-off value for evaluating LH on antagonist administration day was 4.63IU/L (AUC=0.605; P=0.36; **Figure 12**). Using Cumulative CPR as the criterion, the optimal cut-off value for evaluating LH on antagonist administration day was 4.66IU/L(AUC=0.557; P=0.005; **Figure 13**).

## 4 Discussion

Total dosage of Gn in Q4 group was the least, but the number of oocytes retrieved was the most.Although there was no difference in the number of high-quality embryos among the four groups, both Fresh CPR and Cumulative CPR in group Q4 were higher than those in the other three groups.The results were not statistically significant, but they did give us some insight.In predicting the optimal cut-off value of LH on antagonist administration day in multiple pregnancy rates,Almost all the optimal cut-off value are greater than 4, which is similar to the view of Professor Li Yuan’s team (10). Their study concluded that LH≥4IU/L be considered the critical value, and antagonists should be considered when the threshold was exceeded.Our study suggests that fresh ETR would be increased if adding antagonists after LH Level on the antagonist administration day > 4.18IU/L, Cumulative CPR would be increased if adding antagonists after LH Level on the antagonist administration day > 4.66IU/L.

Current research on the relationship between LH levels, ovarian reactivity, and pregnancy outcome during ovulation stimulation has yielded inconclusive results.According to Benmachiche et al. (16) a low LH level on the trigger day was associated with a lower rate of continued pregnancy and live birth and an increased rate of early abortion.Lahoud et al. (6) discovered that mid-follicular LH levels were related to ovarian reactivity but not to live birth rate.Another study (17) found that patients with low basal LH levels (≤3U/L) had no special ovarian responsiveness during ovulation induction but had a poorer pregnancy outcome than those with LH≥3U/L.

LH level is of great significance for maintaining a more appropriate follicle development environment and better receptivity of implanted endometrium in the COH regimen (18, 19). The premature addition of GnRH-ant, excessive use of GnRH-ant, or GnRH-ant usage beyond recommended days may lead to excessive ovarian suppression, thereby resulting in low serum LH levels and a relative lack of estrogen that might affect the growth and development of oocytes. Excessive inhibition of LH level was not found in this study. However, if GnRH-ant is added too late or the dose is insufficient, it may lead to high LH levels and an early LH surge, resulting in decreased follicle quality, reduced pregnancy rate, premature ovulation, and cycle cancellation (20). The increase in serum P level induced by an LH surge can also affect the expression of genes related to endometrial receptivity, thus, affecting embryo implantation (21). Another study reported that an increase in serum P level during the late follicular phase affected not only the embryo quality (22, 23) but also reduced implantation and clinical pregnancy rates (21, 24, 25). However, there are also views that the increase of serum P level at the late follicular stage does not affect embryo quality and cumulative live birth rate (26). This study found that as LH on the antagonist administration day increased, so did LH on the trigger day and P on the trigger day. However, there were no significant differences between the four groups in terms of the number of high-quality embryos and fresh CPR.

Current antagonist protocols are divided into fixed and flexible regimens and are mainly based on Gn stimulation time, follicle development size, and estrogen levels. However, little attention has been paid to LH levels on the antagonist administration day. A study suggested that LH levels can be used as an indicator for the addition of antagonists during COS. Patients with persistently low LH levels (LH<4.0 IU/L) may not require an antagonist (10). It is suggested that the implantation and pregnancy rates decreased with an increased antagonist dosage (27). A study stated that the number of natural killer cells and the expression level of perforin in endometrium were increased in patients treated with GnRH-Ant, hence suggesting that GnRH-ant may reduce endometrial receptivity (28). However, a randomized controlled trial showed that administration of GnRH-ant during the proliferative phase did not affect endometrial receptivity and embryo implantation; the pregnancy rates were not significantly different when compared with controls (29). Studies on the negative effects caused by elevated LH levels have mostly focused on embryo quality and endometrial receptivity. Therefore, the main observation indicators of this study were fresh ETR, fresh CPR,Cumulative CPR,the number of high-quality embryos, and OHSS rate. Although there was no significant difference in the number of high-quality embryos,fresh CPR and Cumulative CPR among the four groups, However, we give the optimal cut-off value of LH on antagonist administration day affecting different pregnancy rates,This has important guiding significance for clinical work.

This is a manuscript with LH on antagonist administration day as the main object of study, and LH on antagonist administration day ‘s significance in pregnancy outcomes of GnRH-ant protocols had not received enough attention before.As this was a retrospective study, many confounding factors limit the generalization of the findings to a certain extent. The sample size is not very large, which weakens the credibility of the study. Additionally, we only included patients undergoing IVF-ET due to female tubal factors, but did not include patients with other common clinical diseases such as PCOS and EMS.In the future, we can consider increasing the sample size for corresponding research.In addition, the lack of studies on abortion rate and live birth rate of pregnancy indicators is also a pity, which can be considered to supplement data based on later follow-up.

## 5 Conclusion

The LH Level on the antagonist administration day could be used as a predictor of the reproductive outcomes in women with normal ovarian function.There was a significant positive correlation between LH on the antagonist administration day and number of oocytes retrieved,number of 2PN embryos, number of blastocysts.LH on the antagonist administration day could predict Fresh CPR, OHSS and Cumulative CPR to some extent.

## Data availability statement

The raw data supporting the conclusions of this article will be made available by the authors, without undue reservation.

## Ethics statement

The studies involving humans were approved by Reproductive Medicine Ethics Committee of the Affiliated Hospital of Shandong University of Chinese Medicine. The studies were conducted in accordance with the local legislation and institutional requirements. The participants provided their written informed consent to participate in this study. Written informed consent was obtained from the individual(s) for the publication of any potentially identifiable images or data included in this article.

## Author contributions

CP contributed to study design, data collection, statistical analysis and drafting of the manuscript. KW assisted with data collection and interpretation and reviewed the analyzed results. HW provided ART-related clinical theory and technical support. CP and KW reviewed the analyzed results and revised the manuscript. All authors contributed to the article and approved the submitted version.
